# Assessment of pre-injury health-related quality of life: a systematic review

**DOI:** 10.1186/s12963-017-0127-3

**Published:** 2017-03-14

**Authors:** Annemieke C. Scholten, Juanita A. Haagsma, Ewout W. Steyerberg, Ed F. van Beeck, Suzanne Polinder

**Affiliations:** Erasmus Medical Center, Rotterdam, The Netherlands

**Keywords:** Wounds and injuries, Health-related quality of life, Pre-injury HRQL, Disability, Retrospective measurement, General population norms

## Abstract

**Background:**

Insight into the change from pre- to post-injury health-related quality of life (HRQL) of trauma patients is important to derive estimates of the impact of injury on HRQL. Prospectively collected pre-injury HRQL data are, however, often not available due to the difficulty to collect these data before the injury. We performed a systematic review on the current methods used to assess pre-injury health status and to estimate the change from pre- to post-injury HRQL due to an injury.

**Methods:**

A systematic literature search was conducted in EMBASE, MEDLINE, and other databases. We identified studies that reported on the pre-injury HRQL of trauma patients. Articles were collated by type of injury and HRQL instrument used. Reported pre-injury HRQL scores were compared with general age- and gender-adjusted norms for the EQ-5D, SF-36, and SF-12.

**Results:**

We retrieved results from 31 eligible studies, described in 41 publications. All but two studies used retrospective assessment and asked patients to recall their pre-injury HRQL, showing widely varying timings of assessments (soon after injury up to years after injury). These studies commonly applied the SF-36 (*n* = 13), EQ-5D (*n* = 9), or SF-12 (*n* = 3) using questionnaires (*n* = 14) or face-to-face interviews (*n* = 11). Two studies reported prospective pre-injury assessment, based on prospective longitudinal cohort studies from a sample of initially non-injured patients, and applied questionnaires using the SF-36 or SF-12. The recalled pre-injury HRQL scores of injury patients consistently exceeded age- and sex-adjusted population norms, except in a limited number of studies on injury types of higher severity (e.g., traumatic brain injury and hip fractures). All studies reported reduced post-injury HRQL compared to pre-injury HRQL. Both prospective studies reported that patients had recovered to their pre-injury levels of physical and mental health, while in all but one retrospective study patients did not regain the reported pre-injury levels of HRQL, even years after injury.

**Conclusions:**

So far, primarily retrospective research has been conducted to assess pre-injury HRQL. This research shows consistently higher pre-injury HRQL scores than population norms and a recovery that lags behind that of prospective assessments, implying a systematic overestimation of the change in HRQL from pre- to post-injury due to an injury. More prospective research is necessary to examine the effect of recall bias and response shift. Researchers should be aware of the bias that may arise when pre-injury HRQL is assessed retrospectively or when population norms are applied, and should use prospectively derived HRQL scores wherever possible to estimate the impact of injury on HRQL.

**Electronic supplementary material:**

The online version of this article (doi:10.1186/s12963-017-0127-3) contains supplementary material, which is available to authorized users.

## Background

Insight into the change from pre- to post-injury health status of trauma patients is important in order to derive population estimates of the impact of injuries on health-related quality of life (HRQL). The difficulty in measuring the impact of injuries is that the patient’s HRQL after sustaining an injury may be influenced by factors other than the injury [1]. For instance, pre-existing comorbidity may contaminate our estimates of the injury-related disability, since HRQL scores might incorporate the impact of one or more comorbid diseases instead of solely reflecting the impact of the injury. To overcome attribution bias (i.e., attributing post-injury HRQL scores solely to the injury when it may have been caused by other factors), information on pre-injury HRQL is vital to make valid estimates of the change from pre- to post-injury HRQL due to the injury under study. However, prospectively collected information on the pre-injury HRQL of injury patients is difficult to obtain.

This has led researchers to use alternative methods to assess the contrast between pre-injury and post-injury HRQL, such as use of patient recall or retrospective baseline scores (in other words, pre-injury HRQL that is assessed after sustaining the injury). However, retrospective baseline scores of pre-injury health status are potentially subject to bias [2, 3]. Patients may remember their pre-injury HRQL as better or worse than it actually was (recall bias) [2]. Moreover, patients’ perception on HRQL may change after the injury, due to a change in internal standards or values (response shift) [4]. This change in perception of HRQL after the injury may also affect the retrospectively assessed pre-injury HRQL.

Other methods are the application of general population norms (i.e., using normative values from the general population as a reference point for the health status before the injury), or the use of a matched non-injured comparison group as a baseline to assess the reduction in health due to the injury. The application of population norms or a matched non-injured comparison group may lead to an inaccurate estimate of the change in health status, as injured people may differ from the general non-injured population [5, 6]. Research indicated that injured people have a higher prevalence of comorbidity, hospitalization, and health service utilization prior to their injury in comparison to non-injured people [5]. This suggests that pre-injury health status is worse compared to population norms and conflicts with the reported better pre-injury health status compared to the general population [6–8]. On the other hand, the injured population might be healthier and more likely to participate in activities, exposing them to a higher risk of injuries [6].

The current systematic review identifies the methods that are used to assess pre-injury health status of trauma patients and to estimate the change from pre- to post-injury HRQL due to an injury. Moreover, bias that may occur from these methods is examined, by comparing the reported pre-injury HRQL scores with population norms by calculating age- and gender-specific norm scores based on the demographics of the included study samples.

The objectives of this study are:

1. To identify the methods which are used to measure pre-injury HRQL;
2. To compare the reported pre-injury HRQL scores with calculated general age- and gender-adjusted norms;
3. To address the pre-injury HRQL scores per HRQL instrument and injury type;
4. To examine the change between pre- and post-injury HRQL in injury patients; and
5. To formulate recommendations for future studies on (pre-injury) HRQL.

## Methods

Relevant studies were identified through systematic literature searches in the databases EMBASE, MEDLINE (via Ovid SP), Cochrane Central, PubMed, Web of Science, SCOPUS, PsycINFO, CINAHL, Lilacs, Scielo, ScienceDirect, and ProQuest. Grey literature was examined via Google Scholar. Search strategies were developed in consultation with a search expert, and included a combination of subheadings and text words (Appendix). Reference lists and citation indices of the included papers and relevant reviews were inspected to identify additional relevant citations.

### Study selection

We included studies that assessed the pre-injury HRQL of injury patients, published in English in peer-reviewed journals until July 6, 2015. We included studies on general injury populations, as well as injury-specific studies (e.g., traumatic brain injury or hip fractures). There was no restriction in the methods of patient selection used in the studies (e.g., samples drawn from the Emergency Department (ED), hospital, or outpatient programs). HRQL was conceptualized as an individual’s perception of how an illness and its treatment affect physical, mental, and social aspects of his/her life [9]. Studies that assessed only some domains of HRQL (e.g., functional status, activities of daily living, mobility, mental health) were excluded. We included studies that assessed the HRQL of patients before the injury, whether assessed before the injury or retrospectively. Studies that solely used population norms, as a substitute of pre-injury HRQL, were excluded. For studies using data from the same study sample, one study was chosen as the reference study by giving priority to the study that focused on reporting pre-injury HRQL summary scores or utility scores (e.g., instead of percentage of problems per HRQL domain).

### Data extraction and methodological quality

The first review author (AS) screened all titles and abstracts and deleted obviously irrelevant papers. Two independent review authors (AS and SP) screened the remaining citations on title and abstract and those obtained in full text. Results from both reviewers were compared by a third review author (JH) and any disagreement was be resolved by discussion between the three authors.

We extracted information on the participants (age and gender), injury (type, severity, and mechanism), the assessment of pre-injury HRQL (instrument, procedure, and timing), and recovery of injury patients (change between pre- and post-injury HRQL).

The methodological quality of the studies was evaluated with four elements of the STROBE checklist [10] which were most relevant to the quality of reported pre-injury HRQL by injury type: setting, participants, data sources/measurement, and study size. In addition, risk of bias was assessed using items from the Research Triangle Institute item bank for observational studies on attrition bias (“Impact missing data adequately assessed”) and reporting bias (“No important primary outcomes missing”) [11].

### Statistical analysis

Pre-injury HRQL scores from the study samples were compared with norm scores derived from the general population. To provide population norms for all studies, we used norms by age and sex groups of the EQ-5D (UK population) [12], SF-36 [13], and SF-12 [14] (both US population) to calculate age- and gender-adjusted norms based on the demographics in the study samples.

Heterogeneity between pre-injury HRQL scores was assessed with the Q-statistic and I^2^-statistic, using a random-effects model in a Microsoft Excel spreadsheet [15]. The Q-statistic is a Chi^2^-test for heterogeneity, which assesses whether observed differences in results are compatible with chance alone. A significant Q (low p-value) indicates heterogeneity among the HRQL scores and a variation that is beyond chance [16]. The I^2^-statistic describes the percentage of variation across studies that is due to heterogeneity rather than chance, with an I^2^ value of 25% or lower is associated with low heterogeneity, 50% indicating substantial heterogeneity, and 75% or higher indicating high heterogeneity [17]. In case of substantial or high heterogeneity, pooled results should not be calculated, or at the very least, should be interpreted with caution.

## Results

### Literature search

**Fig. 1:**
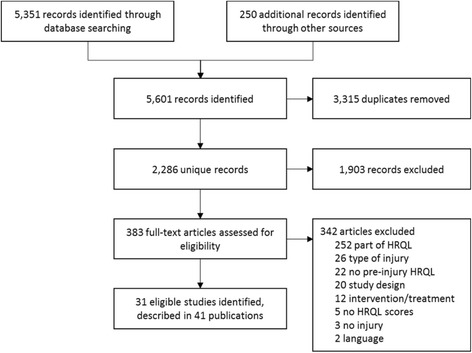
Study selection

The extensive search strategy identified 2,286 unique titles of potentially relevant articles (Fig. 1). Screening of the titles and abstracts resulted in a selection of 383 articles that appeared to meet all selection criteria. After screening and selection of the full text papers, we retrieved 31 studies described in 41 publications. The main reasons for exclusion were not measuring pre-injury health status, not reporting on injuries or only reporting part of the outcomes on HRQL.

### Study characteristics

Of the 31 studies included in our systematic review, most were conducted in the US (*n* = 8) [18–25], Australia (*n* = 5) [6, 26–29], and Canada (*n* = 5) [30–34] (Table 1). Eight studies measured the pre-injury HRQL of patients with a hip fracture [24, 30, 34–39], followed in frequency by extremity injury (*n* = 6) [19, 23, 32, 37, 40, 41], general injury (*n* = 5) [8, 29, 31, 42, 43] and traumatic brain injury (TBI, *n* = 4) [22, 25, 27, 44]. Sample sizes of the studies varied widely, ranging between 34 [33] and 2,842 [8] participants, with most studies having sample sizes between 100 and 600 (*n* = 17). The majority of the participants were males (>50% men in 20 out of the 31 studies). The nine studies that included more women than men [18, 23, 30, 34, 36, 38, 39, 45, 46] often focused on hip fractures (*n* = 5) [30, 34, 36, 38, 39], or reported on the outcomes after a motor vehicle crash of a longitudinal (annual) survey (*n* = 2) [18, 46]. The mean age of the participants in the included studies ranged between 10 [19] and 87 [30], with an average of 30 to 54 in half (*n* = 16) of the studies and 75+ in six of the 31 included studies. Four studies measured the pre-injury HRQL for children and adolescents [19, 22, 25, 31], of which the author names are indicated in bold in Table 1.

**Table 1:** Study characteristics

Author, year, country, design	Type of injury	Setting	Study sample	Instrument	Assessment	Focus of pre-injury and follow-up assessments	Analysis
		Cohort	Inclusion /Exclusion *N* (response); age; % men; severity		Method Timing		Comparison groups
***Alghnam, 2014, US*** [18] ^***A***^	***Motor vehicle injury vs no MVC***	***Medical expenditure panel survey (MEPS)***	***18 + y*** ***N = 993; MVC: 18-45y 68%; 46%*** ***No MVC: 18-45y 51%; 47%***	***SF-12***	***Questionnaires*** ***Pre: prospective*** ***Post: max 9 m***	***NR***	***MVC vs no MVC***
Andrew, 2012, Australia [26] ^A^	Orthopedic injury (sport/recreation)	Trauma services/Hospital (VOTOR)	18-75y *N* = 317 (73%); 40y (13); 73% ISS > 15 19%	SF-36 (v2)	Telephone interviews Pre: 1-2w Post: 12 m	Pre: 4w before injury Post: NR	Type of sport/recreation
Beaupre, 2012, Canada [30] ^A^	Hip fracture	Nursing home facilities	65 + y; Previously ambulatory nursing home residents *N* = 60 (65%); 87y (8), 62–104; 30%	EQ-5D	(Telephone) Interviews Pre: NR Post: 3 m, 6 m, 12 m	Pre: just before injury Post: NR	Survival status
**Brussoni, 2013, Canada** [31] ^D^	General injury	Pediatric ED/hospital	0-16y *N* = 232 (67%); 0-4y 33%, 5-16y 67%; 61% boys	EQ-5D (3 L, VAS)	Questionnaires (child/proxy)	Pre: 1d before injury Post: NR	Length of hospital stay
Buecking, 2014, Germany [35] ^A^	Hip fracture (Proximal femoral)	Hospital/Surgical fracture treatment	>60y; No ISS ≥ 16; No malignancy-related fractures *N* = 350 (65%); 81y (8), 60–99; 73%	EQ-5D (3 L, VAS) German norms	Questionnaires Pre: at admission Post: at discharge	Pre: before injury Post: current status	
Busse, 2012, Canada [32] ^E^	Tibia fracture	Multicenter (SPRINT)	Operative fixation intramedullary nail *N* = 1319; 39y (16); 74%	SF-36	Questionnaires Pre: at the time of enrollment Post: 2w, 12 m	Pre: before injury Post: NR	
**Ding, 2006, US** [19] ^A^	Extremity fracture (Long bone or pelvic)	Pediatric hospital	5-15y, Hospitalized ≥1d; No TBI *N* = 100 (85%); 10y (3); 66%; NISS: 8 (5)	PedsQL	Telephone interviews Pre: soon after injury (median 9d) Post: 3 m, 12 m	Pre: before injury Post: post-injury	Upper/lower extremity; HRQL domains
Dvorak, 2005, Canada [33] ^B,C^	Vertebral fracture (C1, Jefferson)	Databases	18 + y; No neurological injury; Disruption of anterior and posterior atlantal arches *N* = 34 (60%); 48y (21); 68%	SF-36 Canadian norms	Questionnaire (by mail/phone) Not reported	Pre: before injury Post: current status	Canadian norms
Fauerbach, 1999, US [20] ^A^	Burn injury	Burn center	Adults *N* = 86; 42y (15); 78	SF-36	Questionnaire Pre: first 73 h after admission Post: 2 m after discharge	Pre: month before injury Post: past month	Post Traumatic Distress (PTD) vs no PTD; HRQL domains; US norms
Gabbe, 2007, Australia [6] ^A^	Orthopedic injury	Hospital (VOTOR)	18 + y; New orthopedic injury *N* = 1839 (77%); 45y, 21–65; 60%	SF-12	Interviews/Questionnaires Pre: in-hospital/soon after discharge Post: NA	Pre: week before injury Post: NA	Men vs women Australian norms
Greenspan, 2002, US [21] ^A^	Gunshot injury	Hospital	18-64y; <24 h after injury *N* = 60 (38%); 30y (9); 92% ISS 1–9 57%; MAIS 3 48%	SF-36	(Telephone) Interviews Pre: in-hospital/soon after discharge Post: 8 m after discharge	Pre: pre-injury status Post: current status	HRQL domains; UK norms
Griffin, 2015, UK [36] ^A^	Hip fracture	Hospital	60 + y; operatively managed *N* = 741 (83%); 80 + y 67%; 25%	EQ-5D (3 L)	(Telephone) Interviews No response: questionnaires (post) Pre: in-hospital/soon after discharge Post: 4w, 4 m, 12 m	Pre: pre-injury status Post: NR	Age
Gross, 2012, Switzerland [44]^A^	TBI vs no TBI	ICU	≥2 AIS regions, ISS > 16; GCS < 14, AISH > 2 No secondarily admissions *N* = 170 (65%); 40y (21); 75% ISS 28 (8); GCS 11 (5)	EQ-5D (3 L, VAS) German norms SF-36 (v1)	Postal questionnaires Pre & post: 2y	Pre: pre-injury status Post: post-injury status	TBI vs non-TBI
Hagino, 2009, Japan [37]^A^	Hip fracture Vertebral fracture Wrist fracture	Hospital	Women; 45 + y; No malignancy-related fractures; Lower-energy; Minor trauma *N* = 122; Hip 76 (10), 49–91; Vertebral 73 (10), 48–91; Wrist 69 (10), 49-88	EQ-5D Japanese norms	Questionnaires Pre: first visit/admission Post: 2w, 3 m, 6 m, 12 m	Pre: period before injury Post: NR	Hip vs vertebral vs wrist
Innocenti, 2015, Italy [43] ^A^	General injury	ED-HDU	*N* = 153 (51%); 54y (22); 67% ISS 12 (9)	SF-12 Italian norms	Telephone interviews Pre & post: 6 m after ED discharge	Pre: before injury Post: current status	Change HRQL domains Italian norms
Jaglal, 2000, Canada [34] ^E^	Hip fracture	Hospital	Living independently; No cognitive impairment *N* = 43; 81y (8); 19%	SF-36	(Telephone) Interviews Pre: in-hospital Post: 6w, 6 m after injury	Pre: before injury Post: NR	HRQL domains
**Jimenez, 2013, US** [22] ^A^	TBI	Hospital (CHAI)	<18y; discharged alive from ED Hispanic or non-Hispanic white *N* = 531 (73%); 0–9 50%; 65% boys; MAIS 1 46%	PedsQL (Spanish)	(Telephone) Interviews Pre: soon after injury (median 37d) Post: 3 m, 12 m, 24 m, 36 m	Pre: period before injury Post: NR	Hispanic vs non-Hispanic white
Lyrtzis, 2012, Greece [41] ^A^	Ankle sprain (2nd degree)	Not reported	Injury at 1 limb; No previous ankle injury; No fracture; <24 h after injury; no analgesic *N* = 78 (98%); 36y (13); 74%	SF-36	Questionnaires Pre: day of injury Post: 10d	Pre: before injury Post: 10d since injury	HRQL domains
McGuine, 2014, US [23] ^A^	Knee injury	Sports medicine center/clinic	Women; 13-23y; Injury during regular fitness or sport activities *N* = 255 (91%); 17y (2), 13–23; 0%	SF-12 (v2)	Questionnaires Pre: initial visit (median 12d) Post: diagnosis, 3 m, 6 m, 12 m	Pre: 1w before injury Post: since injury	US norms
Ottosson, 2007, Sweden [45] ^A^	Musculoskeletal injury	ED	15 + y *N* = 318 (39%); 39y (15); 46%	SF-36	Questionnaires Pre: at inclusion Post: 1 m, 6 m	Pre: week before injury Post: NR	Swedish norms
Peterson, 2008, US [24] ^E^	Hip fracture	Hospital Hip fracture surgery	>65y; Not mentally impaired; Living independently *N* = 105 (NR); alive – 79 (8); NR	SF-36 (v1)	Questionnaires Pre: NR Post: 1^st^w after operation	Pre: 4w before injury Post: NR	Survival status; HRQL domains
**Pieper, 2014, US** [25] **Related:** [60] ^A^	TBI vs no TBI	Pediatric ED (Self-selected sample)	5-17y; discharge <24 h *N* = 40 mBTI, 40 no TBI TBI: 12y (3); 80%; no TBI: 10y (3); 63%	PedsQL (4.0 Generic)	(Telephone) Interviews Pre: initial contact Post: 1 m, 3 m, 6 m, 12 m	Pre: week before injury Post: NR	mTBI vs no TBI vs no injury; Children vs parents (proxy); HRQL domains
Ponsford, 2011, Australia [27] ^A^ Related: [61]	TBI vs no TBI	Hospital	18 + y; <24 h after injury *N* = 123 (63%) mTBI, 100 (30%) no TBI mTBI: 35y (13); 74%; no TBI: 35y (11); 64%	SF-36	Questionnaires Pre: within 48 h after injury Post: 1w, 3 m	Pre: before injury Post: current, past 4w	mTBI vs no TBI
***Pons-Villanueva, 2011, Spain*** [46] ^***A***^	***Motor vehicle injury vs no MVC***	***University graduates (SUN)***	***N = 64 MVC, 3297 no MVC (91%)*** ***40y; 38%***	***SF-36***	***Questionnaires*** ***Pre: prospective*** ***Post: 4y, 8y***	***NR***	***MVC vs no MVC;*** ***HRQL domains***
Skoog, 2001, Sweden [40]	Tibia shaft fracture	Hospital	No pathologic fractures or fractures adjacent to implant *N* = 64; 45y (19), 14–93; 56%	SF-36	Interviews/Questionnaires Pre: during hospitalization Post: 4 m, mean 13 m	Pre: before injury Post: not reported	Swedish norms
Sugeno, 2008, Japan [38] ^A^	Hip fracture	Hospital	No severe cognitive decline *N* = 50 (44%); 77y (10); 20%	EQ-5D (3 L, VAS)	Interviews Pre: 1/2d after admission Post: discharge, 3 m, 6 m, 12 m after admission	NR	
Tidermark, 2002, Sweden [39] ^A^	Hip fracture (Falls)	ED	65 + y; Living independently *N* = 90; 80y (7), 66–92; 37%	EQ-5D (3 L, VAS) UK norms	Interviews/Questionnaires (post) Pre: first days after injury Post: 1w, 4 m, mean 17 m (2))	Pre: week before injury Post: NR	Age (60–88); Gender; Fracture outcome; Survival status; Swedish norms
Ulvik, 2008, Norway [42] ^A^	General injury	Closed ICU (neurosurgery)	>18y *N* = 210 (92%); 39y (17), 18–83; 81% ISS (median) 25; 4-54	EQ-5D	Telephone interviews Pre & post: 2-7y (median 4y)	Pre: before injury Post: current status	
Wasiak, 2014, Australia [28] ^A^	Burn injury	Burn center	18 + y; TBSA > 10% *N* = 99 (79%); 42y (2); 75%	SF-36 (v2)	Questionnaires Pre: not reported Post: 12 m	NR	Australian norms
Watson, 2005, Australia [29] ^A^ Related: [7]	General injury	Hospital	18-74y; No self-inflicted injury; No neurological deficit *N* = 221 (88%); 38y; 72%	SF-36	Interviews Pre: in hospital / 1^st^w Post: 6w, 3 m, 6 m, 12 m	Pre: previous week Post: previous week	Age; Gender; Work status; Employment; Australian norms
Wilson, 2012, New Zealand [8] ^A^ Related: [62–64]	General injury	Accident Compensation Corporation entitlement claims register	18-64y; No self-harm or sexual assault *N* = 2.842; 18-34y 35%, 35-64y 47%; 61%	EQ-5D New Zealand norms	Interviews Pre: 3.2 m Post: 4.6 m, 12.3 m	Pre: before injury Post: current status	Recovery status; New Zealand norms

(Bold author names are studies of children; Studies in bold and italics prospectively measured pre-injury HRQL)

Design: ^a^ Prospective cohort, ^b^ Retrospective cohort, ^c^ Cross-sectional, ^d^ Validation study, ^e^ Randomized controlled trial

*h* hour, *d* day, *w* week, *m* month, *y* year, *AIS* abbreviated injury scale, *ED* emergency department, *GCS* Glasgow Coma Scale, *NR* not reported, *MVC* injury due to motor-vehicle crash, *Ortho* orthopedic injury, *TBI* traumatic brain injury

### Methodological quality

Over half (*n* = 19) of the 31 articles included in our review reported on attrition. Most studies faced several problems in the participation of eligible patients, as patients refused to participate (*n* = 15), could not be contacted (*n* = 6), did not complete the HRQL assessment (*n* = 6), had died (*n* = 5), or were not able to respond to the questionnaires (e.g., due to the consequences of the trauma, *n* = 3). Overall, response rates ranged from 60 to 98% in 17 of the 22 studies that reported on response rates.

Limited variation existed in the selection of samples between the studies. Most patients were recruited during or after a treatment in a (pediatric) hospital (*n* = 21), while others were selected from a specialized burn center (*n* = 2) [20, 28], sports center (*n* = 1) [23], or nursing home facility (*n* = 1) [30].

In four out of the 31 studies, the measurement of pre-injury HRQL was one of the primary aims [8, 18, 46], while in all other studies pre-injury HRQL scores were used to assess the change in HRQL after the injury or to validate HRQL instruments.

### Methods to measure pre-injury HRQL

The 36-item Short-Form (SF-36, *n* = 14) [21, 24, 26–29, 32–34, 40, 41, 45–47] was the most frequently used instrument to assess the pre-injury HRQL of injury patients, followed by the EuroQol-5 Dimension Questionnaire (EQ-5D, *n* = 9) [8, 30, 31, 35–39, 42], and the SF-12 (*n* = 4) [6, 18, 23, 43] (Table 1). The remaining studies used the Pediatric Quality of Life Inventory (PedsQL, *n* = 3) [19, 22, 25], or a combination of the EQ-5D and SF-36 (*n* = 1) [44]. The majority of the studies assessed the participants’ pre-injury HRQL by using a questionnaire (*n* = 16) [18, 20, 22–24, 27, 28, 31–33, 35, 37, 41, 44–46] or a face-to-face interview (*n* = 11) [8, 21, 25, 29, 30, 34, 36, 38–40]. At follow-up, most studies used questionnaires (*n* = 17) [18, 23, 24, 27, 28, 31–33, 35, 37, 39–41, 44–47] followed by the use of telephone interviews (*n* = 10) [19, 21, 22, 25, 26, 30, 34, 36, 42, 43].

All but two studies in this review retrospectively assessed the pre-injury HRQL of patients, by asking them to recall their HRQL before the injury occurred. Only two studies provided prospectively collected pre-injury health status of participants [18, 46] (articles in bold and italics in Table 1): the Medical Expenditure Panel Survey (MEPS) [18] and the Seguimiento Universidad de Navarra (SUN) [46] cohort. These studies used data from longitudinal cohort studies in which participants who were initially non-injured were followed for several years, by means of questionnaires comprising the SF-36 [46] or SF-12 [18]. In addition, only one of the included studies measured the recalled pre-injury health status of trauma patients and not their post-injury HRQL [6], while all other studies measured both pre- and post-injury HRQL.

Pre-injury scores were often reported as assessed “soon after” injury or admission (*n* = 12), in-hospital or “soon after” discharge (*n* = 5) [21, 29, 34, 36], at inclusion or initial contact/visit (*n* = 5) [23, 25, 32, 37, 45], within six months after ED discharge (*n* = 2) [8, 43], or years after injury (*n* = 2) [42, 44]. The focus of the questionnaire and/or interviews (i.e., a specified period prior to the injury) was often not specifically defined (e.g., “before the injury,” *n* = 15) or not reported (*n* = 2) [28, 38]. The studies that specified the period of their pre-injury assessment used a day before injury (*n* = 1) [31], “just” before injury (*n* = 1) [30], a week before injury (*n* = 4) [25, 39, 45], the previous week (*n* = 2) [23, 29], or the month or four weeks before injury (*n* = 3) [20, 24, 26].

Most studies (*n* = 16) made a comparison of pre-injury HRQL between injury patients or with controls (e.g., TBI vs. no TBI) [18, 19, 25, 27, 37, 44, 46], between subgroups (e.g., by age, gender, ethnicity) [6, 22, 29, 36, 39], or between survival or recovery status (e.g., survived vs. dead, recovered vs. not recovered) [8, 24, 30, 39]. In addition, twelve studies compared the participants’ pre-injury health status with general population norms [8, 20, 21, 23, 28, 29, 33, 39, 40, 43, 45].

### Comparison of pre-injury HRQL between injury patients and with population norms

Within-study comparisons between retrospectively collected pre-injury HRQL and general population norms indicated that self-reported pre-injury HRQL scores were consistently higher than population norm scores (*n* = 3) [8, 29, 48]. Three studies found that scores were higher in either the physical domains (*n* = 3) [20, 21, 28] or mental domains (*n* = 1) [20] or in certain age or sex groups (*n* = 1) [6]. Five studies found no differences between the recalled pre-injury HRQL and population norms (*n* = 5) [33, 39, 40, 43, 45] (Table 2).

**Table 2:** Pre- and post-injury HRQL

Author, year, country	Instrument	Pre-injury HRQL	Post-injury HRQL	Change	Findings
				Post-injury vs pre-injury	
General injury					
**Brussoni, 2013, Canada** [31]	EQ-5D	Not admitted: 0.97 1-3d: 0.94 4 + d: 0.93	Not admitted: 0.90 1-3d: 0.76 4 + d: 0.61	Not admitted: −0.07 1-3d: −0.18 4 + d: −0.32	All categories of length of stay in hospital had significantly lower HRQL at follow-up than at baseline
Ulvik, 2008, Norway [42]	EQ-5D	0.97	0.70	−0.27^a^	Significant decrease in HRQL in all dimensions
Wilson, 2012, New Zealand [8]	EQ-5D	0.94	5 m: 0.75 12 m: 0.78	5 m: −0.19 12 m: −0.16	Significantly higher pre-injury HRQL than New Zealand norms. Recovered had significantly higher post-injury HRQL than norms. Non-recovered had significantly lower HRQL than norms.
Watson, 2005, Australia [29]	SF-36	PCS 55; MCS 55	1w: PCS 25; MCS 46 6w: PCS 34; MCS 53 12w: PCS 38; MCS 55 26w: PCS 43; MCS 52 52w: PCS 44; MCS 52	1w: PCS −30; MCS −9 6w: PCS −21; MCS −2 12w: PCS −17; MCS 0 26w: PCS −12; MCS −3 52w: PCS −11; MCS −3	Consistently higher pre-injury scores than Australian norms. Males had higher pre-injury PCS and MCS than females. 18-24y and 65-74y had highest pre-injury MCS. Those with pre-injury paid-employment had significantly higher pre-injury PCS than those without.
Innocenti, 2015, Italy [43]	SF-12	PCS 53 (7), 24–64 MCS 55 (7), 28-63	6 m: PCS 41 (12), 14–64 6 m: MCS 46 (13), 16-67	PCS −12^a^ MCS −9^a^	93% pre-injury PCS and MCS in normal range according to Italian norms. Significant worse HRQL after 6 m.
Traumatic brain injury					
Gross, 2012, Switzerland [44]	EQ-5D SF-36	TBI: 99 (4); no TBI: 95 (14) *TBI* PCS 57 (6); MCS 50 (11) *no TBI* PCS 56 (7); MCS 51 (12)	TBI: 65 (28); no TBI: 76 (21) TBI - PCS: 44 (12); MCS: 39 (13) *no TBI* - PCS: 45 (11); MCS: 48 (13)	TBI: −34; no TBI: −19 *TBI* PCS −13; MCS −11 *no TBI* PCS −11; MCS −3	TBI had significantly worse HRQL compared with no TBI (on EQ VAS, EQ-5D, MCS, but not on PCS)
Ponsford, 2011, Australia [27]	SF-36	*mTBI* PCS 54 (6); MCS 49 (8) *no TBI* PCS 54 (6); MCS 53 (7)	1w: *mTBI* PCS: 38 (10); MCS: 44 (11) 1w: *no TBI* PCS:36 (10); MCS: 49 (11) 3 m: *mTBI* PCS: 52 (9); MCS: 48 (10) 3 m: *no TBI* PCS: 50 (9); MCS: 53 (7)	1w: *mTBI* PCS: −16; MCS: −5 1w: *no TBI* PCS: −18; MCS: −4 3 m: *mTBI* PCS: −2; MCS: −1 3 m: *no TBI* PCS: −4; MCS: 0	mTBI had significantly poorer mental HRQL pre-injury. Significant change in PCS in mTBI and no TBI, MCS only in mTBI. Scores dropped dramatically at 1w, returned to pre-injury levels at 3 m.
**Jimenez, 2013, US** [22]	PedsQL	NHW: 86 Hispanic: 90	NR	0-3 m: NHW −5; Hispanic −16 0-12 m: NHW −5; Hispanic −13 0-24 m: NHW −5; Hispanic −13 0-36 m: NHW −5; Hispanic −16	Pre-injury scores were higher for Hispanic than NHW. Post-injury scores were significantly lower for Hispanic compared with NHW.
**Pieper, 2014, US** [25]	PedsQL	mTBI: 82 (13) no TBI: 81 (14)	mTBI: 82 (15) no TBI: 82 (16)	mTBI 0 no TBI: 1	No significant differences were identified among mTBI, NBI, and uninjured groups. Cognitive HRQL after mTBI trended lower from 3–12 months post-injury.
Hip fracture					
Beaupre, 2012, Canada [30]	EQ-5D	0.62 (0.20) *Survived* 0.63 (0.20) *Deceased* 0.61 (0.20)	*Survivors* 3 m: 0.42 (0.25) 6 m: 0.46 (0.24) 12 m: 0.42 (0.30)	3 m: −0.21 6 m: −0.17 12 m: −0.21	At 1y, those alive had higher pre-injury HRQL than those that died. Significant loss in HRQL at 3 m that remained relatively unchanged 6 m and 12 m postoperatively.
Buecking, 2014, Germany [35]	EQ-5D	0.71	Discharge: 0.46	Discharge: −0.25	Significantly reduced HRQL during hospitalization.
Griffin, 2015, UK [36]	EQ-5D^b^	0.56	4w: 0.28 4 m: 0.32 12 m: 0.36	4w: −0.28 4 m: −0.24 12 m: −0.2	Significantly lower HRQL at one year than pre-injury. HRQL significantly improved after 4w in those aged ≤80y, but not in >80y.
Hagino, 2009, Japan [37]	EQ-5D	0.80 (0.17)	2w: 0.37 (0.27) 3 m: 0.64 (0.16) 6 m: 0.63 (0.18) 12 m: 0.68 (0.24)		Hip fracture had lower pre-injury HRQL than wrist facture (significant) or vertebral fracture.
Sugeno, 2008, Japan [38]	EQ-5D	0.77 (0.24)	Discharge: 0.67 (0.21) 12 m: 0.81 (0.17)	Discharge: −0.10 12 m: 0.04	HRQL decreased post-injury, but recovered to pre-facture levels 1y following hospitalization.
Tidermark, 2002, Sweden [39]	EQ-5D	0.78 (0.21) *Survived* 0.79 (0.21) *Deceased* 0.73 (0.22)	*Survivors* 1w: 0.44 (0.33) 4 m: 0.55 (0.37) 12 m: 0.51 (0.36)	1w: −0.34 4 m: −0.23 12 m: −0.27	Similar pre-injury HRQL compared to Swedish population norms. Decrease in HRQL from pre- to post-injury. Patients did not regain their pre-injury HRQL.
Jaglal, 2000, Canada [34]	SF-36	PF 74 (24); RP 68 (46); BP 92 (16); GH 79 (20); VT 63 (22); SF 86 (21); RE 86 (34); MH 73 (20)	6w: PF 44 (18); RP 2 (7); BP 68 (20); GH 75 (19); VT 54 (18); SF 75 (23); RE 85 (36); MH 79 (16) 6 m: PF 59 (22); RP 63 (48); BP 78 (24); GH 77 (25); VT 59 (23); SF 77 (25); RE 96 (21); MH 82 (13)	6w: PF −30^a^; RP −66^a^; BP −24^a^; GH −4; VT −9; SF −11^a^; RE −1^a^; MH 6 6 m: PF −15^a^; RP −5^a^; BP −14^a^; GH −2; VT −4; SF −9; RE 10; MH 9^a^	Significant decrease in HRQL from pre- to post-injury in all domains (ex GH, VT, MH). Significantly lower PF, RP, BP but higher MH at 6 m than pre-injury.
Peterson, 2008, US [24]	SF-36	*Survived* PF 56 (36); RP 81 (33); BP 84 (24); GH 75 (21); VT 65 (22); SF 86 (23); RE 93 (26); MH 76 (20) *Died* PF 41 (29); RP 60 (43); BP: 82 (24); GH 62 (26); VT 55 (23); SF 84 (24); RE 85 (32); MH 79 (22)	NA		At recruitment, no differences in domain scores between those living at 5 years and those dead (though small N, large SD). At 5y, significantly higher PF, RP and GH in those alive than those that died.
Extremity injury					
**Ding, 2006, US** [19] **(Extremity)**	PedSQL	89	3 m: 73 12 m: 80	3 m: −16 12 m: −9	Similar pre-injury HRQL for upper- and lower- extremity fractures. Significantly lower HRQL post-injury than pre-injury.
Busse, 2012, Canada [32] (Tibia)	SF-36	PCS 53 (9) MCS 54 (9)	2w: PCS 28 (8); MCS 46 (13) 12 m: PCS 43 (11); MCS 52 (12)	2w: PCS −25; MCS −8 12 m: PCS −10; MCS −2	Decrease in HRQL from pre- to post-injury. Patients did not regain their pre-injury HRQL.
Skoog, 2001, Sweden [40] (Tibia)	SF-36^b^	PF 72; RP 83; BP 80; GH 80; VT 75; SF 83; RE 88; MH 82	4 m: PF 60; RP 45; BP 63; GH 74; VT 62; SF 70; RE 58; MH 77 12 m: PF 68; RP 58; BP 66; GH 70; VT 57; SF 70; RE 76; MH 73	4 m: PF −12^a^; RP −38^a^; BP −17; GH −6; VT −13; SF −13^a^; RE −30^a^; MH −5 12 m: PF −4; RP −25; BP −14^a^; GH −10^a^; VT −18^a^; SF −13; RE −12; MH −9	Pre-injury HRQL was comparable to Swedish healthy population. SF-36 domain scores were lower at 4 m and 12 m, compared to pre-injury HRQL.
Lyrtzis, 2012, Greece [41] (Ankle)	SF-36	89 (6); 68–97 PF 96; RP 95; BP 91; GH 76; VT 79; SF 92; RE 93; MH 87	10d: 68 (11); 52–82 PF: 64; RP: 72; BP: 71; GH: 54; VT: 78; SF: 77; RE: 82; MH: 68	10d: −21 PF −32; RP −23; BP −20; GH −22; VT −1; SF −15; RE −11; MH −19	Significant worsening of HRQL 10d after injury, compared to pre-injury HRQL.
McGuine, 2014, US [23] (Knee)	SF-12	PCS 56 (5) MCS 56 (7)	Diagnosis: PCS 41 (11); MCS 51 (12) 3 m: PCS 48 (9); MCS 53 (10) 6 m: PCS 53 (7); MCS 53 (9) 12 m: PCS 54 (6); MCS 54 (8)	Diagnosis: PCS −15; MCS −5 3 m: PCS −8; MCS −3 6 m: PCS −3; MCS −3 12 m: PCS −2; MCS −2	Pre-injury HRQL was higher than population norms in all domains. HRQL change from preinjury through an entire 12 m after injury.
Hagino, 2009, Japan [37] (Wrist)	EQ-5D	0.93 (0.13)	2w: 0.72 (0.14) 3 m: 0.81 (0.18) 6 m: 0.87 (0.15) 12 m: 0.88 (0.15)	2w: −0.21 3 m: −0.12 6 m: −0.06 12 m: −0.05	Hip fracture had lower pre-injury HRQL than wrist facture (significant) or vertebral fracture. Scores showed recovery after 6 m. After 1y, scores were not significantly different from pre-fracture.
Other injury					
***Pons-Villanueva, 2011, Spain*** [46] ***(MVC)***	SF-36	*MVC* PCS 53; MCS 47 PF 95; RP 87; BP 74; GH 73; VT 65; SF 89; RE 80; MH 71 *No MVC* PCS 53; MCS 49 PF 95; RP 91; BP 79; GH 76; VT 66; SF 92; RE 87; MH 76	*MVC* PCS 51; MCS 48 PF 93; RP 83; BP 69; GH 71; VT 63; SF 91; RE 82; MH 73 *No MVC* PCS 53; MCS 50 PF 95; RP 92; BP 78; GH 77; VT 66; SF 94; RE 90; MH 77	*MVC* PCS −2; MCS 1 PF −2; RP −4; BP −5; GH −2; VT −2; SF −2; RE 2; MH 2 *No MVC* - PCS 0; MCS 1 PF 0; RP 1; BP −1; GH 1; VT 0; SF 2; RE 3; MH 1	All physical scales declined in participants reporting a MVC, while mental health dimensions increased. Patients who did not have any MVC had significantly higher HRQL than those who suffered a MVC on RP, BP, GH, RE, MH, MCS and PCS.
***Alghnam, 2014, US*** [18] ***(MVC)***	SF-12	*MVC* PCS 50; MCS 49 *No MVC* PCS 50; MCS 51	*MVC* PCS 47; MCS 49 *No MVC* PCS 50; MCS 51	*MVC* PCS −3; MCS 0 *No MVC* PCS 0; MCS 0	Similar baseline PCS in MVC and no MVC. Significant lower baseline MCS in MVC than no MVC.
Ottosson, 2007, Sweden [45] (Muscosk)	SF-36^b^	Recovered 1 m: PF 93; RP 93; BP 92; GH 85; VT 75; SF 92; RE 94; MH 85 Not recovered: PF 85; RP 83; BP 80; GH 83; VT 73; SF 91; RE 83; MH 85	1 m: *Rec 1 m* PF 95; RP 93; BP 89; GH 85; VT 73; SF 95; RE 93; MH 85 *No rec* PF 63; RP 30; BP 43; GH 68; VT 45; SF 68; RE 85; MH 67 6 m: *No rec* PF 70; RP 45; BP 53; GH 65; VT 51; SF 76; RE 60; MH 70	1 m: *Rec 1 m* PF 2; RP 0; BP −3; GH 0; VT −2; SF 3; RE −1; MH 0 *No rec* PF −22; RP −53; BP −37; GH −15; VT −28; SF −23; RE −30; MH −18 6 m: *No rec* PF −15; RP −38; BP −27; GH −18; VT −22; SF −15; RE −23; MH −15	Pre-injury HRQL was comparable to Swedish norm population. At 1 m patients who reported no recovery had significantly lower scores on all domains, compared to those reporting recovery.
Andrew, 2012, Australia [26] (Ortho)	SF-36	PCS 59 (4); MCS 55 (7) PF 57 (3); RP 56 (4); BP 60 (6); GH 60 (6); VT 60 (8); SF 56 (5); RE 55 (5); MH 55 (7)	PCS 52 (10); MCS 53 (10) PF 52 (8); RP 50 (10); BP 52 (10); GH 55 (10); VT 52 (10); SF 52 (10); RE 53 (7); MH 52 (9)	PCS −7; MCS −2 PF −5; RP −6; BP −7; GH −5; VT −7; SF −4; RE −2; MH −3	Significant reductions in all SF-36 subscale scores, with RP and BP reporting the most reductions.
Gabbe, 2007, Australia [6] (Ortho)	SF-12	PCS 51; Men 53; Women 48 MCS 55; Men 55; Women 54	NA		Significantly higher PCS (stratified men 25-54y) and MCS (men 18-24y, women 18-24y, 25-34y or 45-54y) than Australian norms.
Dvorak, 2005, Canada [33] (Spine)	SF-36	PCS 49 (13) MCS 52 (10)	PCS 43 (13) MCS 49 (14)	PCS −6 MCS −3	No significant differences between patients’ recalled PCS and MCS and Canadian norms.
Hagino, 2009, Japan [37] (Spine)	EQ-5D	0.88 (0.17)	2w: 0.53 (0.17) 3 m: 0.76 (0.18) 6 m: 0.75 (0.16) 12 m: 0.84 (0.17)	2w: −0.35 3 m: −0.12 6 m: −0.13 12 m: −0.04	Hip fracture had lower pre-injury HRQL than wrist (significant) or vertebral fracture. Scores at 6 m were significantly lower than pre-injury. After 1y, scores were not significantly different from pre-fracture values.
Fauerbach, 1999, US [20] (Burn)	SF-36	*PTD* PF 87 (24); RP 85 (34); BP 87 (28); GH 77 (25); VT 66 (20); SF 88 (24); RE 85 (32); MH 77 (14) *No PTD* PF: 92 (20); RP 91 (22); BP 81 (30); GH 87 (11); VT: 73 (20); SF 94 (19); RE 97 (16); MH 88 (9)	2 m: *PTD* PF 66 (27); RP 29 (39); BP 41 (19); GH 68 (24); VT 52 (24); SF 75 (30); RE 76 (38); MH 67 (22) *No PTD* PF 85 (22); RP 56 (49); BP 47 (21); GH 83 (15); VT 69 (23); SF 92 (18); RE 92 (34); MH 87 (12)	2 m: *PTD* PF −21; RP −56; BP −46; GH −9; VT −14; SF −13; RE −9; MH −10 *No PTD* PF −7; RP −35; BP −34; GH −4; VT −4; SF −2; RE −5; MH −1	Higher pre-injury HRQL in PTD (BP) and non-PTD (MH, VT, RE, SF, GH) than US norms.
Wasiak, 2014, Australia [28] (Burn)	SF-36	PCS 56 (9) MCS 52 (12)	PCS 52 (13) MCS 52 (11)	PCS −4 (1) MCS 0 (1)	Pre-burn PCS was higher than Australian norms, MCS was comparable. HRQL at 12 m were consistent with the Australian norms. Significant lower PCS at 12 m compared with pre-injury.
Greenspan, 2002, US [21] (Gunshot)	SF-36	PF 96 (14); RP 89 (29); BP 93 (19); GH 85 (20); VT 70 (21); SF 86 (27); RE 83 (34); MH 76 (24)	8 m: PF 71 (28); RP 43 (42); BP 63 (32); GH 58 (27); VT 52 (28); SF 67 (31); RE 64 (43); MH 68 (25)	PF −25; RP −46; BP −30; GH −27; VT −18; SF −19; RE −19; MH −8	Pre-injury scores were similar to population norms, except for PF and GH (higher). Significant declines in PCS and MCS, and across all domains compared to pre-injury (especially PF, RP, BP, GH, and VT).

(Bold author names are studies of children; Studies in bold and italics prospectively measured pre-injury HRQL)

^a^Significant change between pre- and post-injury HRQL scores

^b^Scores obtained from graph(s) (not reported in text or tables)

**Fig. 2:**
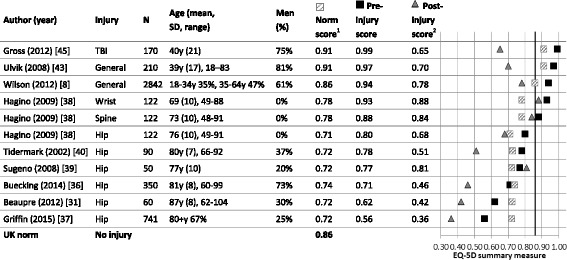
Pre-injury EQ-5D scores by injury type and in comparison to population norm scores. ^1^Adjusted by the age and sex distribution in the study population, based on the weighted health state index by age and sex [12]. ^2^Final post-injury measurement: at discharge [35], 1 year post-injury [8, 30, 36–39], 2 years post-injury, or 2–7 years post-injury [42]

**Fig. 3:**
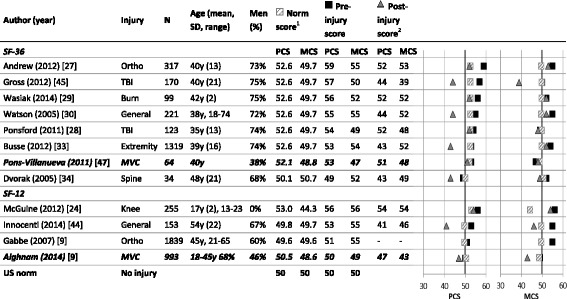
Pre-injury SF-36 and SF-12 scores by injury type and in comparison to population norm scores. ***Studies in bold and italics prospectively measured pre-injury HRQL***. ^1^ Adjusted by the age and sex distribution in the study population, based on the weighted health state index by age and sex [13, 14]. ^2^ Final post-injury measurement: at 3 [27], 6 [43] or ***maximal 9 months post-injury*** [18], 1 year post-injury [26, 28, 29, 32, 33, 48], 2 years post-injury [44], or ***4***–***8 years post-injury*** [46]. Heterogeneity: PCS Chi^2^ = 12.48, df = 11, (*p* = 0.33), I^2^ = 12%; MCS Chi^2^ = 11.88, df = 11, (*p* = 0.37), I^2^ = 7%. MVC: injury due to motor vehicle crash; Ortho: orthopedic injury; TBI: traumatic brain injury

The self-reported pre-injury HRQL scores also exceeded the calculated age- and gender-adjusted population norm scores on the EQ-5D [8, 37–39, 42, 44] (Fig. 2), as well as the physical and mental domains of the SF-36 and SF-12 (Fig. 3). Exceptions were injury types of higher severity, including elderly hip fracture patients (aged 80+ years) [30, 35, 36], or patients with a motor vehicle injury [18, 46], vertebral fracture [33], or TBI [27].

Within-study comparisons of pre-injury HRQL between injury patients or with controls showed that patients who were injured due to a motor vehicle injury or who sustained a TBI had significantly lower mental health at baseline [18, 27, 44, 46] and lower scores across all HRQL domains [46] compared to those without a motor-vehicle injury or TBI (Table 2). Higher pre-injury HRQL was found in those who survived than those who eventually died during follow-up (significant differences found on the SF-36 PF, RP and GH [24], no significant differences found between EQ-5D scores [30]) and in those recovered than those not recovered at follow-up (not significant) [8].

### Pre-injury HRQL scores per HRQL instrument and injury type

There was a large variation in the presentation of the pre-injury HRQL of patients (Table 2). Most studies reported the total scale scores on the EQ-5D (*n* = 10) [8, 30, 31, 35–39, 42, 44] or PedsQL (*n* = 3) [19, 22, 25]. The studies that used the SF-36 or SF-12 often presented the physical (PCS) and mental component scores (MCS) (*n* = 10) [18, 27–29, 32, 33, 43, 44, 48], while some studies provided an oversight of all domain scores without summary scores [20, 21, 24, 34, 41].

Pre-injury HRQL scores varied between patients with a hip fracture, ranging from 0.56 in an operatively managed sample of primarily 80 + −year-old females [36] to 0.80 in a hospitalized sample of women aged 45+ [37]. Highest pre-injury EQ-5D scores were seen in study populations who experience a TBI [44], major trauma [42], unintentional injury [8], or wrist or vertebral fracture [37] (mean EQ-5D 0.94, SD 0.04) while lowest pre-injury EQ-5D scores were reported in hip fracture populations [30, 35–39] (mean EQ-5D 0.71, SD 0.10); two-sample t(9) = 5.01, 95% confidence interval (CI) [0.13–0.34], *p* = 0.001. Overall, pre-injury EQ-5D scores decreased with age, from 0.99 in populations with a mean age of 40 years (SD 21) [44] to 0.56 in those aged 80+ years [30, 35, 36].

Patients with a vertebral injury reported lowest pre-injury PCS (SF-36, PCS 49) [33], while those with orthopedic injury reported highest pre-injury PCS scores (SF-36, PCS 59) [26]. Lowest pre-injury MCS on both the SF-36 (MCS 47) [46] and SF-12 (MCS 49) [18] was reported in the two studies that prospectively assessed the pre-injury HRQL of participants before the occurrence of a motor-vehicle injury. Overall, rather similar pre-injury HRQL scores were reported in all studies, showing low heterogeneity (PCS: I^2^ = 12%, MCS: I^2^ = 7%), with generally better pre-injury PCS than MCS (mean 54.6 vs 52.9).

### Change between pre- and post-injury HRQL

Most studies used a longitudinal design (*n* = 23) with multiple follow-up measurements over time (*n* = 18), often measuring post-injury HRQL at three months, six months and/or 12 months. All studies showed a decrease in post-injury HRQL compared to their pre-injury levels of HRQL (Table 2). Looking at the EQ-5D, only one out of the 12 studies showed full recovery to pre-injury HRQL at one year after the injury [38], while the other studies still reported reduced levels of HRQL post-injury. Looking at the SF-36 and SF-12, injuries showed to have the highest impact on the physical component of HRQL (reduction in PCS with 15 to 30 points from pre-injury to first post-injury assessment) compared to the mental component of HRQL (reduction in MCS with 5 to 9 points) [23, 27, 29, 32]. At the final follow-up measurement, both prospective studies showed almost full recovery to pre-injury HRQL levels on the PCS and full recovery on the MCS [18, 46], while only one retrospective study showed such recovery on the PCS [48] or MCS [28].

## Discussion

This systematic review summarized the methods that were used to assess pre-injury health status and to estimate the change from pre- to post-injury HRQL. All but two of the 31 studies in our review used retrospective assessment (recall) to assess pre-injury HRQL. The studies most often applied the SF-36, followed by the EQ-5D or SF-12, by means of questionnaires or face-to-face interviews. Recalled pre-injury HRQL scores consistently exceeded general population norms, except in a limited number of studies on injury types of higher severity (e.g., traumatic brain injury and hip fractures). All studies reported reduced post-injury HRQL compared to pre-injury HRQL. Both prospective studies reported that patients had recovered to their pre-injury levels of physical and mental health, while in all but one retrospective study patients had not returned to their reported pre-injury levels of HRQL, even years after the injury.

Prospective assessment is the preferred method to determine pre-injury HRQL as it is not subject to bias that may occur due to experiencing an injury. In our review, only two out of the 31 studies used prospective assessment of pre-injury HRQL. These studies used longitudinal data from the Medical Expenditure Panel Survey (MEPS) among the US general population [18] and the Seguimiento Universidad de Navarra (SUN) cohort comprising university graduates in Navarra, Spain [46]. Both prospective studies reported lowest pre-injury mental health on the SF-36 (MCS 47) [46] as well as SF-12 (MCS 49) [18] of all studies in our review, which otherwise all used retrospective assessment. These prospective studies indicate that the retrospective assessment and population norm approach are highly likely to be biased.

Our review shows that the retrospectively assessed pre-injury HRQL systematically differed from the age- and gender-adjusted norms we calculated based on population data on the EQ-5D, SF-36, and SF-12. Despite the use of different HRQL instruments, recalled pre-injury HRQL scores in our review consistently exceeded these adjusted population norms. An exception to this were samples including patients with a hip fracture [30, 35, 36, 39], motor vehicle injury [18, 46], vertebral fracture [33] or TBI [27], that reported poorer pre-injury HRQL than our calculated adjusted norms. These injury patients are likely to be less healthy than their counterparts [18, 27, 44, 46], in terms of socioeconomic status [18], comorbidity [18, 49], or frailty and older age [12, 49, 50].

The difference between retrospectively assessed pre-injury HRQL and population norm scores might be caused by several reasons.

Recall bias may have influenced the outcomes of the retrospective assessment, as patients may have remembered their pre-injury HRQL differently than it actually was [2, 51, 52]. Patients may, for example, have overestimated their health status before the injury, resulting in higher recalled pre-injury HRQL than seen in the general population.

Response shift might have occurred, as patients’ perception of HRQL may have changed due to the injury and a change in health [4]. After having had experience with poor HRQL, patients may have inflated the rating of their health status before the injury [53].

Nevertheless, some researchers argue for the use of retrospective assessment of pre-injury HRQL, as this method applies one internal standard of HRQL values (reference point) in the assessment of both pre-injury HRQL and post-injury HRQL [4, 53]. According to them, such a reference point is essential for the interpretation of the change from pre- to post-injury HRQL, since patients may have changed their judgement of HRQL due to new insights since the injury (e.g., although a patient has a serious injury, he/she has seen others who are far worse off), or patients have become used to their new health state. However, both recall bias and response shift might result in an overestimation of the pre-injury HRQL by patients. This is underpinned by our finding that, even years after the injury, in all but one retrospective study patients had not returned to their reported levels of pre-injury PCS and MCS, while recovery to pre-injury HRQL levels was seen in both prospective studies.

Moreover, selection bias may have threatened the validity of the findings from the studies included in our review, as the study populations were often not randomly selected from the injury population for which the findings are reported [54]. For example, studies had excluded patients with pre-existing morbidities (e.g., physical illness, cognitive impairment), as it was anticipated that these patients would be difficult to follow up. Exclusion of patients with impairments before the injury may have increased the overall pre-injury HRQL scores of these study samples, as healthier participants were recruited.

In contrast, attrition bias may have decreased the overall pre-injury HRQL scores measured in the studies, as a higher proportion of the non-participants were less educated [26], cognitively impaired [38], victim of intentional injury [6], shorter hospitalized [21] and had lower injury severity [28, 29, 44], less pain [34], better mental health [34]. These factors are all expected to be associated with better HRQL and incorporation of these patients may have resulted in higher pre-injury HRQL scores. Additionally, pre-injury HRQL levels may have increased after loss of follow up, resulting in higher pre-injury HRQL in the final study sample with complete response compared to the eligible study sample [32].

Finally, retrospectively assessed pre-injury HRQL scores may differ from the population norms as injury populations may differ from the general population. The findings of the retrospective assessments (recall) in our review suggest that injured populations are generally healthier than the general population. Previous studies reported that, as injured populations might be healthier, they are more likely to participate in activities, exposing them to a higher risk of injuries [6]. However, the comparisons of injury patients with matched controls in our review showed injury patients to be less healthy than their counterparts, as they reported significantly lower pre-injury mental health than controls [18, 27, 44, 46] and lower scores across all HRQL domains [46]. Previous research showed that injury patients had a higher occurrence of comorbidity, higher admission rates to the hospital, higher health service utilization, and a lower socioeconomic status prior to their injury in comparison to uninjured people [5, 18]. It is argued that the general population has not been exposed to a similar injury experience as the injury population, which emphasizes the use of retrospective assessments over the application of general population norms to estimate the impact of injury on HRQL [7].

### Strengths and limitations

Our review included studies on the pre-injury HRQL from children, adolescents, and adult patients, with various injury types, using a range of HRQL instruments. Moreover, this review compared the reported pre-injury HRQL scores with general population norms, calculated for each study based on the reported mean age and gender distribution of the study sample, to identify bias that may occur from the different methods to assess pre-injury HRQL.

There are limitations to this review that need to be addressed. First, there was no restriction in the methods of patient selection used in the studies. Therefore, the studies in this review included samples retrieved from a variety of injury settings (e.g., hospital or outpatient programs). Their conclusion may not be applicable to injury patients from other injury settings. However, most studies selected their patients during or after treatment in a (pediatric) hospital or specialized treatment center, which may enhance the generalizability of their results to patient populations with similar case mix.

Second, the review included studies with patient samples from a broad range of injury types and injury severity levels, which may have complicated the comparability of the results between studies. Nonetheless, this way we were able to provide a full oversight of the pre-injury health status of injury patients and the differences in pre-injury HRQL between injury types.

In addition, there are limitations to the studies included in our review. First, more than half of the included studies had difficulties in recruiting research participants, as patients often could not be contacted, had died, refused to participate, or did/could not complete questionnaires. The studies often reported limited generalizability of their results due to differences between the eligible patients and study participants, loss to follow-up, their limited number of subjects, and recruitment of participants from a single center.

In some studies pre-injury HRQL was assessed after a long period of time since the injury, for example several months up to years after the injury [8, 42, 44]. This longer time frame may have increased the recalled pre-injury HRQL scores [31], as these studies also reported the highest pre-injury HRQL scores on the EQ-5D (0.94–0.99) compared to the studies that used shorter time frames. However, these three studies assessed the HRQL of a relatively young injury population. Moreover, no differences were found between the time frame and pre-injury HRQL in studies that used the SF-36 or SF-12.

Finally, unfortunately not all studies reported the HRQL scores in the text or tables (e.g., only in graphs). After contacting the authors, in three publications HRQL scores had to be manually obtained from the graphs presented in the article [36, 40, 45]. This may have resulted in some small differences in the levels of pre- and/or post-injury HRQL.

### Recommendations for future research

Our review clearly showed that recalled pre-injury HRQL systematically exceeded population norms. These differences in pre-injury HRQL may generate different estimates of the change in HRQL from pre- to post-injury due to an injury.

Researchers should use prospectively derived pre-injury HRQL scores wherever possible to estimate the impact of injury on HRQL. If it is not feasible to prospectively assess the pre-injury health status of trauma patients, researchers should be aware of the bias that may arise when pre-injury HRQL is assessed retrospectively or when population norms are applied. Overall, more research is needed to examine the effect of recall bias and response shift on the reported levels of pre-injury HRQL among trauma patients, in which different methods to assess pre-injury HRQL are compared and within-study comparisons between reported pre-injury HRQL and population norms are made.

The results of our review imply that there are a number of methodological advances regarding pre-injury HRQL interpretation left. Researchers should be aware of the different purposes the information on pre-injury HRQL of patients may have. For instance, pre-injury HRQL may be seen as a baseline health status to which patients are expected to return after the injury. On the other hand, pre-injury HRQL may be used to measure total loss in health, or may be used to offer insight into inter-patient differences in recovery after an injury.

In general, when assessing pre-injury HRQL, researchers should carefully consider and specify the timing of the assessment of pre-injury HRQL and the period of the pre-injury assessment. The time period shows to be one of the essential factors influencing patient recall, as recall bias is generally worse when asking for a recall over longer periods [55]. A short time frame within the injury and retrospective assessment of pre-injury HRQL may increase recall and may increase the correlation between pre- and post-injury measures [31]. This implies that pre-injury HRQL should be assessed as soon as possible after the injury, preferably within the first week after the injury [56]. Whether or not the measurement of pre-injury HRQL is the primary purpose of studies, publications on the measurement of HRQL should include information on the applied methods to measure HRQL.

Levels of pre-injury HRQL also may have been influenced by the use of telephone interviews. In our review, the highest or one of the highest pre-injury HRQL on the EQ-5D [42], SF-36 (PCS and MCS) [26], or SF-12 [43] were reported by studies that had conducted telephone interviews to assess the pre-injury levels of HRQL. Previous research indicated that telephone-administered questionnaires provide higher HRQL scores than self-administered questionnaires [57–59]. Preferably, the same method should be used for the assessment of both pre-injury and post-injury HRQL throughout the study, at all post-injury HRQL measurements and among all individuals.

Researchers should choose a validated HRQL instrument that has shown good performance in the type of injury under study, and that is sensitive to changes in HRQL and differentiate well between health states. In order to assess the change from pre- to post-injury HRQL, the same HRQL instrument should be applied throughout the study. Preferably, a HRQL instrument should be chosen for which national age- and gender-adjusted population norms are available. In order to enable comparison of the impact of injuries on HRQL between studies, injury types and other diseases, it is recommended to report the pre- and post-injury HRQL scores for specific age and sex groups, which correspond to the age and sex distribution of the norm groups for the applied instrument.

Finally, to examine the change in HRQL due to the injury, a longitudinal design is recommended with multiple follow-up measurements over time (e.g., at 1–3 months, 3–6 months, and 6–24 months post-injury) [56].

## Conclusions

So far, primarily retrospective research has been conducted to assess pre-injury HRQL. This research shows consistently higher pre-injury HRQL scores than population norms and a recovery that lags behind that of prospective assessments, implying a systematic overestimation of the change in HRQL from pre- to post-injury due to an injury. More prospective research is necessary to examine the effect of recall bias and response shift. Researchers should be aware of the bias that may arise when pre-injury HRQL is assessed retrospectively or when population norms are applied, and should use prospectively derived HRQL scores wherever possible to estimate the impact of injury on HRQL.

## Appendix

### Literature search strategies

**Table 3:** Date: July 6, 2015

Database	Records	Unique records
Embase	1360	1343
MEDLINE (Ovid)	1151	289
Web of Science	882	117
SCOPUS	1001	88
CINAHL (EBSCO)	511	143
PsycINFO (Ovid)	240	53
Cochrane Library	44	0
PubMed	13	11
LILACS	0	0
SciELO	0	0
ScienceDirect	91	78
ProQuest	58	14
Google Scholar	250	150
Total	5601	2286

**Embase 1360**

(‘quality of life’/exp OR ‘health status’/de OR ‘health disparity’/de OR ‘health status indicator’/de OR ‘functional status’/de OR ‘daily life activity’/exp OR ‘general health status assessment’/exp OR (‘quality of life’ OR ‘life quality’ OR HRQL OR HRQoL OR qol OR ((health OR functional* OR psych*) NEAR/3 (status*)) OR (daily NEAR/3 (life OR living) NEAR/3 activit*) OR adl OR badl OR iadl OR ((baseline OR preinjur* OR ‘pre-injury’) NEAR/3 (health OR disabilit* OR status*)) OR ((pre OR before) NEAR/3 (injur* OR trauma* OR accident* OR traffic*) NEAR/3 (health OR disabilit*))):ab,ti) AND ((preinjur* OR ((pre OR before) NEXT/3 (injur* OR trauma* OR accident* OR traffic* OR fracture*)) OR pretraum*):ab,ti)

**MEDLINE (Ovid) 1151**

(‘quality of life”/ OR “health status”/ OR “Health Status Disparities”/ OR “Health Status Indicators”/ OR “Sickness Impact Profile”/ OR “Activities of Daily Living”/ OR (“quality of life” OR “life quality” OR HRQL OR HRQoL OR qol OR ((health OR functional* OR psych*) ADJ3 (status*)) OR (daily ADJ3 (life OR living) ADJ3 activit*) OR adl OR badl OR iadl OR ((baseline OR preinjur* OR “pre-injury”) ADJ3 (health OR disabilit* OR status*)) OR ((pre OR before) ADJ3 (injur* OR trauma* OR accident* OR traffic*) ADJ3 (health OR disabilit*))).ab,ti.) AND ((preinjur* OR ((pre OR before) ADJ3 (injur* OR trauma* OR accident* OR traffic* OR fracture*)) OR pretraum*).ab,ti.)

**Web of Science 882**

TS = (((“quality of life” OR “life quality” OR HRQL OR HRQoL OR qol OR ((health OR functional* OR psych*) NEAR/2 (status*)) OR (daily NEAR/2 (life OR living) NEAR/2 activit*) OR adl OR badl OR iadl OR ((baseline OR preinjur* OR “pre-injury”) NEAR/2 (health OR disabilit* OR status*)) OR ((pre OR before) NEAR/2 (injur* OR trauma* OR accident* OR traffic*) NEAR/2 (health OR disabilit*)))) AND ((preinjur* OR ((pre OR before) NEAR/2 (injur* OR trauma* OR accident* OR traffic* OR fracture*)) OR pretraum*)))

**SCOPUS 1001**

TITLE-ABS-KEY(((“quality of life” OR “life quality” OR HRQL OR HRQoL OR qol OR ((health OR functional* OR psych*) W/2 (status*)) OR (daily W/2 (life OR living) W/2 activit*) OR adl OR badl OR iadl OR ((baseline OR preinjur* OR “pre-injury”) W/2 (health OR disabilit* OR status*)) OR ((pre OR before) W/2 (injur* OR trauma* OR accident* OR traffic*) W/2 (health OR disabilit*)))) AND ((preinjur* OR ((pre OR before) W/2 (injur* OR trauma* OR accident* OR traffic* OR fracture*)) OR pretraum*)))

**CINAHL (EBSCO) 511**

(MH “quality of life + ” OR MH “health status + ” OR MH “Health Status Disparities + ” OR MH “Health Status Indicators + ” OR MH “Sickness Impact Profile + ” OR MH “Activities of Daily Living + ” OR (“quality of life” OR “life quality” OR HRQL OR HRQoL OR qol OR ((health OR functional* OR psych*) N3 (status*)) OR (daily N3 (life OR living) N3 activit*) OR adl OR badl OR iadl OR ((baseline OR preinjur* OR “pre-injury”) N3 (health OR disabilit* OR status*)) OR ((pre OR before) N3 (injur* OR trauma* OR accident* OR traffic*) N3 (health OR disabilit*)))) AND ((preinjur* OR ((pre OR before) N3 (injur* OR trauma* OR accident* OR traffic* OR fracture*)) OR pretraum*))

**PsycINFO (Ovid) 240**

(“quality of life”/ OR “Activities of Daily Living”/ OR (“quality of life” OR “life quality” OR HRQL OR HRQoL OR qol OR ((health OR functional* OR psych*) ADJ3 (status*)) OR (daily ADJ3 (life OR living) ADJ3 activit*) OR adl OR badl OR iadl OR ((baseline OR preinjur* OR “pre-injury”) ADJ3 (health OR disabilit* OR status*)) OR ((pre OR before) ADJ3 (injur* OR trauma* OR accident* OR traffic*) ADJ3 (health OR disabilit*))).ab,ti.) AND ((preinjur* OR ((pre OR before) ADJ3 (injur* OR trauma* OR accident* OR traffic* OR fracture*)) OR pretraum*).ab,ti.)

**Cochrane Library 44**

((‘quality of life’ OR ‘life quality’ OR HRQL OR HRQoL OR qol OR ((health OR functional* OR psych*) NEAR/3 (status*)) OR (daily NEAR/3 (life OR living) NEAR/3 activit*) OR adl OR badl OR iadl OR ((baseline OR preinjur* OR ‘pre-injury’) NEAR/3 (health OR disabilit* OR status*)) OR ((pre OR before) NEAR/3 (injur* OR trauma* OR accident* OR traffic*) NEAR/3 (health OR disabilit*))):ab,ti) AND ((preinjur* OR ((pre OR before) NEXT/3 (injur* OR trauma* OR accident* OR traffic* OR fracture*)) OR pretraum*):ab,ti)

**PubMed 13**

(“quality of life”[mh] OR “health status”[mh] OR “Health Status Disparities”[mh] OR “Health Status Indicators”[mh] OR “Sickness Impact Profile”[mh] OR “Activities of Daily Living”[mh] OR (“quality of life” OR “life quality” OR HRQL OR HRQoL OR qol OR health status*[tiab] OR functional status*[tiab] OR psychological status*[tiab] OR (daily AND (life OR living) AND activit*[tiab]) OR adl OR badl OR iadl) OR ((pre OR before) AND (injur*[tiab] OR trauma*[tiab] OR accident*[tiab] OR traffic*[tiab]) AND (health OR disabilit*[tiab])))) AND ((preinjur*[tiab] OR pre injur*[tiab] OR pre trauma*[tiab] OR pretraum*[tiab])) AND publisher[sb]

**LILACS 0**

**SciELO 0**

(“quality of life” OR HRQL OR HRQoL OR qol OR “health status” OR “functional status” OR “psychological status” OR “daily life activities” OR “activities of daily living” OR “baseline health” OR “baseline disability” OR “baseline status” OR “preinjury health” OR “preinjury disability”) AND (“pre injury” OR “pre trauma” OR “pre accident” OR “pre traffic” OR “before injury” OR “before trauma” OR “before accident” OR “before traffic” OR preinjury)

**ScienceDirect 91**

(“quality of life” OR HRQL OR HRQoL OR qol OR “health status” OR “functional status” OR “psychological status” OR “daily life activities” OR “activities of daily living” OR “baseline health” OR “baseline disability” OR “baseline status” OR “preinjury health” OR “preinjury disability”) AND (“pre injury” OR “pre trauma” OR “pre accident” OR “pre traffic” OR “before injury” OR “before trauma” OR “before accident” OR “before traffic” OR preinjury) AND LIMIT-TO(topics, “injury”).

**ProQuest 58**

(ti(“quality of life” OR HRQL OR HRQoL OR qol OR “health status” OR “functional status” OR “psychological status” OR “daily life activities” OR “activities of daily living” OR “baseline health” OR “baseline disability” OR “baseline status” OR “preinjury health” OR “preinjury disability”) OR ab(“quality of life” OR HRQL OR HRQoL OR qol OR “health status” OR “functional status” OR “psychological status” OR “daily life activities” OR “activities of daily living” OR “baseline health” OR “baseline disability” OR “baseline status” OR “preinjury health” OR “preinjury disability”)) AND (ti(“pre injury” OR “pre trauma” OR “pre accident” OR “pre traffic” OR “before injury” OR “before trauma” OR “before accident” OR “before traffic” OR preinjury) OR ab(“pre injury” OR “pre trauma” OR “pre accident” OR “pre traffic” OR “before injury” OR “before trauma” OR “before accident” OR “before traffic” OR preinjury))

**Google Scholar 250**

“quality of life”|HRQL|HRQoL|qol|“health|functional|psychological status”|“daily life|living activities”|“baseline|preinjury health|disability|status” “pre|before injury|trauma|accident|traffic”|preinjury

## Additional file

Additional file 1: Data_Assessment of pre-injury health-related quality of life: a systematic review. (XLSX 59 kb)

## Abbreviations

EQ-5D: EuroQol-5 Dimension Questionnaire
HRQL: Health-related quality of life
MCS: Mental component score
MEPS: Medical Expenditure Panel Survey
PCS: Physical component score
PedsQL: Pediatric Quality of Life Inventory
SF-36: 36-item Short-Form
SUN: Seguimiento Universidad de Navarra
TBI: Traumatic brain injury
